# Antimicrobial peptide AR‐23 derivatives with high endosomal disrupting ability enhance poly(l‐lysine)‐mediated gene transfer

**DOI:** 10.1002/jgm.3259

**Published:** 2020-08-28

**Authors:** Shi‐Kun Zhang, Lin Gong, Xue Zhang, Zhi‐Min Yun, Su‐Bo Li, Hong‐Wei Gao, Cong‐Jie Dai, Jian‐Jun Yuan, Jing‐Ming Chen, Feng Gong, Ying‐Xia Tan, Shou‐Ping Ji

**Affiliations:** ^1^ Department of Stem Cell and Regenerative Medicine Institute of Health Service and Transfusion Medicine Beijing China; ^2^ College of Oceanology and Food Sciences Quanzhou Normal University Quanzhou Fujian China; ^3^ PLA navy No. 971 Hospital Qingdao Shandong China; ^4^ Quanzhou Preschool Education College Quanzhou Fujian China

**Keywords:** antimicrobial peptides, endosomal release, gene transfection, Glu replacement, pH‐sensitive peptides, poly(l‐lysine) (PLL)

## Abstract

**Background:**

pH‐sensitive peptides are a relatively new strategy for conquering the poor endosomal release of cationic polymer‐mediated transfection. Modification of antimicrobial peptides by exchanging positively‐charged residues with negatively‐charged glutamic acid residues (Glu) greatly improves its lytic activity at the endosomal pH, which could improve cationic polymer‐mediated transfection.

**Methods:**

In the present study, we investigated the effect of the number of Glu substituted for positively‐charged residues on the endosomal escape activity of AR‐23 and the ability of mutated AR‐23 with respect to enhancing cationic polymer‐mediated transfection. Three analogs were synthesized by replacing the positively‐charged residues in the AR‐23 sequence with Glu one‐by‐one.

**Results:**

The pH‐sensitive lysis ability of the peptides, the effect of peptides on the physicochemical characteristics, the intracellular trafficking, the transfection efficiency and the cytotoxicity of the polyplexes were determined. Increased lytic activity of peptides was observed with the increased number of Glu replacement in the AR‐23 sequence at acidic pH. The number of Glu substituted for positively‐charged residues of AR‐23 dramatically affects its lysis ability at neutral pH. Triple‐Glu substitution in the AR‐23 sequence greatly improved poly(l‐lysine)‐mediated gene transfection efficiency at the same time as maintaining low cytotoxicity.

**Conclusions:**

The results indicate that replacement of positively‐charged residues with sufficient Glu residues may be considered as a method for designing pH‐sensitive peptides, which could be applied as potential enhancers for improving cationic polymer‐mediated transfection.

## Graphical abstract

Designing pH‐sensitive peptides by replacement of positively‐charged residues with sufficient Glu residues, which could disrupt the lysosome membrane at acidic pH, could be applied as a potential enhancer for improving cationic polymer‐mediated transfection.

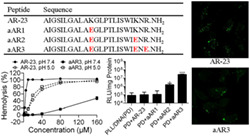

## 1 INTRODUCTION

Non‐viral gene delivery vectors are potential alternatives to viral vectors because of their reduced immunogenicity, low toxicity and easy producibility, despite their lower capacity of gene transfection compared to that of viral vectors.^1^, ^2^, ^3^, ^4^ A major drawback limiting the transfection efficiency of non‐viral gene delivery vectors has historically been the poor release of DNA from endosomal compartments.^5^, ^6^ Given the challenges associated with traditional gene delivery methodology, there is clearly a need for gene delivery technology to enable endosomal escape with minimal cytotoxicity. Strategies to achieve endosomolysis have traditionally been based on osmotic agents, fusogenic lipids and fusogenic peptides.^7^, ^8^, ^9^ The addition of pH‐sensitive peptides has been shown to potentially enhance gene expression of non‐viral gene delivery vectors.^10^, ^11^

Melittin, a 26‐residue peptide from the venom of *Apis mellifera* honeybees, is a well‐studied endosomal disrupting peptide.^12^, ^13^ Melittin or its analogues have been incorporated into polyplex formulations to increase transfection efficiency in different cell lines.^7^, ^14^, ^15^ Melittin, covalently attached to polyethyleneimine (PEI), enables the efficient release of PEI/DNA polyplexes from endosomes, increasing its nuclear localization and subsequently enhancing the transfection activity of PEI/DNA polyplexes in a broad range of cell lines.^16^ However, inherent lytic activity at neutral pH also provokes high cytotoxicity as a result of cell membrane damage. Increasing negatively‐charged residues could also improve the endosomal escaping activity of melittin. For example, acidic modification of melittin by exchanging neutral glutamines (Gln25 and Gln26) with negatively‐charged Glu greatly improved its lytic activity at the endosomal pH of 5.0 with lower lytic activity at neutral condition.^17^ Moreover, Ahmad *et al*.^18^ modified membrane disrupting antimicrobial peptides, LL‐37, melittin and bombolitin V, by replacing all positively‐charged residues with glutamic acid. These analogs are pH‐sensitive and cause endosomal disrupting activity with insignificant cytotoxicity at pH 7.4.^18^ We also designed endosomolytic peptides by replacing the positively‐charged residues with Glu in melittin and RV‐23, an antimicrobial peptide (AMP) from *Rana draytonii*. The designed peptides showed pH‐sensitive lytic activity, which promotes endosomal release of PEI/DNA polyplexes. The incorporation of pH‐sensitive peptides into polyplexes enhanced the PEI‐mediated transfection efficiency, corresponding to up to 42‐fold higher luciferase activity compared to that of PEI alone.^11^ The results indicate that replacement of positively‐charged residues with glutamic acid residues in the AMP sequence yields pH‐sensitive peptides, which enhance the transfection efficiency of PEI/DNA polyplexes.

AR‐23, a frog skin‐derived AMP from *Rana tagoi*, is a melittin related peptide which shows 81% sequence identity.^19^, ^20^ Furthermore, AR‐23 has similar positively‐charged residues distribution pattern at the positions 1, 8 and 17 position to melittin and RV‐23. The present study aimed to investigate the effect of the number of glutamic acids substituted for positively‐charged residues on the endosomal escape activity of AR‐23 and the ability of modified AR‐23 for enhancing cationic polymer‐mediated transfection. Because PEI itself has endosomal disrupting activity as a result of the ‘proton sponge effect’,^21^ poly(l‐lysine) (PLL), a cationic polymer with high DNA condense ability but poor endosome release activity, was used to investigate the ability of the modified peptide to enhance cationic polymer‐mediated transfection.^22^ One to three positively‐charged residues (lysine or arginine) of AR‐23 were replaced by Glu, respectively. To explore the molecular basis of their pH‐sensitive cellular toxicity, we investigated the membrane lytic activity of the derived peptides by hemolytic tests and calcein acetoxymethyl ester (Calcein‐AM) assays. The transfection efficiency of nanoparticles containing PLL/DNA with or without the peptides was investigated and compared with the transfection efficiency of the commercial reagent Lipofectamine 2000 in different cell lines.

## 2 MATERIALS AND METHODS

### 2.1 Chemicals

PLL (molecular weight, 15–30 kDa) and 4',6‐diamidino‐2‐phenylindole dihydrochloride (DAPI) were purchased from Sigma (Taufkirchen, Germany), and PLL was dissolved in double‐distilled water at 5 mg/ml as a stock solution. High glucose Dulbecco's modified Eagle's medium (DMEM), fetal bovine serum (FBS), Opti‐MEM medium and Lipofectamine 2000 were obtained from Invitrogen Corp. (Carlsbad, CA, USA). 3‐[4,5‐dimethylthiazol‐2‐yl]‐2,5‐diphenyltetrazolium bromide (MTT) was obtained from Amresco (Solon, OH, USA). Fluorescein isothiocyanate (FITC)‐labelled oligodeoxynucleotide (FITC‐ODN), 21 nucleotides with a random sequence, was purchased from RiboBio (Guangzhou, China). Calcein‐AM was purchased from Meilunbio (Dalian, China). The luciferase assay system (E1501) and pGL3‐Control plasmids (*Photinus pyralis* luciferase under the control of the SV40 promoter) were obtained from Promega (Madison, WI, USA). pCMV‐N‐EGFP plasmid was purchased from Beyotime Biotechnology (Shanghai, China). The BCA protein assay kit was obtained from Thermo Fisher Scientific Inc. (Rochester, NY, USA).

### 2.2 Peptide synthesis

Peptides (Table 1) were synthesized using the standard Fmoc procedure purified by reverse‐phase (RP) semi‐preparative high‐performance liquid chromatography (HPLC) and were dissolved in dimethylsulfoxide (DMSO) to yield a 1000 μm stock solution for further use. The purity of the synthetic peptide was greater than 95%. The purity of the peptides was verified by analytical RP‐HPLC and was further characterized by mass spectrometry in electrospray positive ion detection mode using an Agilent 1100 ESI/MS system (Agilent Technologies, Santa Clara, CA, USA).

**TABLE 1:** The amino acid sequence and physical characteristics of the peptides

Peptide	Sequence	Calculated mass MW ^a^	Observed mass MW ^a^	Net charge	Total hydrophobic moment^b^
AR‐23	AIGSILGALAKGLPTLISWIKNR.NH_2_	2391.93	2391.95	3	5.18
aAR1	AIGSILGALAEGLPTLISWIKNR.NH_2_	2392.87	2392.89	1	6.38
aAR2	AIGSILGALAEGLPTLISWIENR.NH_2_	2393.81	2393.83	–1	5.33
aAR3	AIGSILGALAEGLPTLISWIENE.NH_2_	2366.74	2366.76	−3	7.62

^a^ Molecular weight (MW) as measured by mass spectrometry.

^b^ Total hydrophobic moment determined by MPEx.

### 2.3 Circular dichroism (CD) spectropolarimetry of the peptides

CD spectra were recorded using a JASCO J‐715 spectropolarimeter (JASCO Inc., Easton, MD, USA) in a 0.1‐cm path length cell under nitrogen at 25°C. The spectra were recorded between 190 and 250 nm with a peptide concentration of 50 μm in 50% trifluoroethanol (TFE) at pH 7.4 and pH 5.0. The percentage of α‐helix structure was calculated as:

$$\alpha \%=100\times \frac{\theta_{222}}{\theta_{f}}\;\text{and}\;\theta_{f}=-39500\times \frac{1-2.57}{N}$$

where α is the amount of helix, *n* is the number of amino acid residues, and [θ]_222_ is the experimentally observed absolute mean residue ellipticity at 222 nm.^23^

### 2.4 Cell culture

Human cervix carcinoma (HeLa) cells, human embryonic kidney 293 (HEK293) cells, human astroglioma cells (U251) and SV40 transformed African Green Monkey kidney cell line (COS7) from ATCC (Manassas, VA, USA) were maintained in our laboratory. The three cell lines were cultured in DMEM supplemented with 10% (v/v) FBS, 2 mm l‐glutamine, 100 U/ml penicillin and 100 U/ml streptomycin unless specified otherwise. The cells were trypsinized using trypsin‐ethylenediaminetetraacetic acid and maintained in a humidified incubator with 5% CO_2_ at 37°C.

### 2.5 Hemolytic activity of the peptides

The study protocol and acquisition of blood samples were approved by the Tissue Committee and Research Ethics Board, Institute of Health Service and Transfusion Medicine, Beijing, China. Hemolytic activity of the peptides was assayed by standard procedures with slight modification.^24^ Fresh human erythrocytes were washed three times with PBS and resuspended at 1.25% hematocrit in either 150 mm NaCl, 20 mm Hepes at pH 7.4, or 150 mm NaCl, 15 mm citric acid at pH 5.0. Then, 160 μl of resuspended erythrocytes was added to a V‐bottom 96‐well plate (Corning Inc., Lowell, MA, USA). Forty microlitres of peptides dissolved in either pH 7.4 buffer solution or pH 5.0 buffer solution, as described above, was then added. After incubation at 37°C for 30 minutes, the samples were centrifuged, and the absorbance of the supernatant was measured at 450 nm using a multiwell microplate reader (SpectraMax M5; Molecular Devices, Sunnyvale, CA, USA). For negative and positive controls, human red blood cells (hRBCs) in either pH 7.4 or 5.0 buffers without peptide (*A*_blank_) and in 0.1% (v/v, final concentration) Triton X‐100 (*A*_triton_) were used, respectively. The percentage of hemolysis was calculated according to:

$$\text{Hemolysis}\;\left(\%\right)=\frac{A_{\text{sample}}-A_{\text{blank}}}{A_{\text{triton}}-A_{\text{blank}}}\times 100\%$$

### 2.6 Assay for hRBCs membrane integrity

CaAM, a non‐fluorescent cell‐permeable derivative of calcein, becomes fluorescent upon hydrolysis by intracellular esterases in live cells, which are retained in the cytoplasm.^25^ Accordingly, the release of caAM can be used to assess peptide‐induced cell membrane permeabilization. For this purpose, hRBCs were incubated with 8 μm caAM in PBS with 0.6% hematocrit for 1 hour, and then free caAM and calcein were removed by washing the cells three times with PBS. The hRBCs were then resuspended in either pH 7.4 solution or pH 5.0 buffer. The indicated peptides were subsequently added (10, 20, and 40 μm), followed by 30 minutes of incubation at 37°C. The fluorescence intensities of calcein were measured with a multiwell microplate reader (SpectraMax M5; Molecular Devices) in fluorescence mode using excitation and emission wavelengths of 490 and 515 nm, respectively. The percentage of calcein release was calculated as follows^26^:

$$\text{Calcein release}\;\left(\%\right)=\frac{F_{p}-F_{c}}{F_{p}-F_{t}}\times 100\%$$

where *F*_p_ is the fluorescence of the sample containing the peptide, *F*_c_ is the fluorescence of the sample without the peptide and *F*_t_ is the fluorescence of the sample after the addition of Triton X‐100 (0.1% final concentration).

### 2.7 Preparation of polyplexes

All the PLL/DNA and PLL/peptide/DNA polyplexes were freshly prepared before use. Polyplexes were prepared by adding PLL solution to equal volumes of plasmid solution (final N/P ratio of 5, which determines the ratio of nitrogen (N) in the PLL to phosphate (P) in the nucleic acid) and incubated for 15 minutes at room temperature. Then, the solution was added to the same volume of the indicated amounts of peptides and incubated for 15 minutes at room temperature before characterization and gene transfection experiments. Polyplexes for physicochemical characterization and transfection experiments were prepared in double‐distilled water and serum‐free DMEM, respectively.

### 2.8 Intracellular trafficking of polyplexes

The cellular uptake efficiency was evaluated in HeLa cells. Briefly, HeLa cells (1.5 × 10^4^ cells/well) were seeded in a CVG 8‐well chamber (NUNC Lab‐Tek; Thermo Fisher Scientific Inc.). Polyplexes were prepared as above and using 50 nm FITC‐ODN instead of 8 μg/ml plasmid DNA. Twenty‐four hours later, the medium was replaced with 300 μl of polyplexes. After incubating at 37°C for 1 hour, the cells were washed three times with PBS and then incubated with serum‐free DMEM containing 50 nm Lyso‐Tracker Red DND‐99 for 1.5 hour. The cells were then washed three times with PBS and fixed with 4% paraformaldehyde. The cells were stained with the nuclear stain DAPI (final concentration 300 nm) for 5 minutes at room temperature and observed under a confocal laser scanning microscope using a 63 × objective (CLSM; LSM880; Carl Zeiss, Oberkochen, Germany).

### 2.9 Physicochemical characterization of polyplexes

#### 2.9.1 DNA gel retardation

To test the effect of peptides on the DNA condensation ability of PLL, agarose gel electrophoresis was performed.^27^ Ten microliters of DNA, 10 μl of PLL/DNA or 10 μl of PLL/DNA/peptide polyplexes were mixed with 10 × loading buffer such that the amounts of DNA were identical (0.2 mg DNA/well). The samples were then loaded into the slots of a 0.3% agarose gel containing 0.5 mg/ml ethidium bromide. Electrophoresis was carried out at 120 V for 30 minutes in the 1 × TAE running buffer. DNA retardation was analyzed using a gel image system (model 1600; Tanon, Shanghai, China) to indicate the location of the DNA.

#### 2.9.2 Characterization of particle sizes and zeta potential

The size and zeta potential of the polyplexes were evaluated via laser light scattering using a Malvern NANO‐ZS90 (Malvern Instruments, Malvern, UK).

### 2.10 Transfection assay

HeLa cells, HEK293 cells, U251 cells and COS7 cells were separately seeded in 96‐well plates (Corning Inc., Lowell, MA, USA) at a density of 5 × 10^3^ cells/well on the day before transfection. When confluence reached 70–80%, the cells were transfected with PLL/DNA or PLL/DNA/peptide polyplexes containing 200 ng of DNA and incubated for 4 hours. The medium was then changed with fresh DMEM growth medium, and the cells were incubated for the reporter gene assays. Transfection mediated by Lipofectamine 2000 was performed in accordance with the manufacturer's instructions. Luciferase gene expression was measured at 24 hours after transfection. Luciferase assays were performed with a Luciferase assay system, and relative light units (RLU) were measured with a 96 Microplate Luminometer (Glomax; Promega). Protein concentrations in the cell extracts were measured by a BCA assay. The final values were calculated as RLU/mg protein and reported as the mean ± SD obtained from triplicate transfections. HEK293 cells was used to evaluate the transfection of PLL/DNA/peptide polyplexes with pCMV‐N‐EGFP plasmid. Images were taken using the DMI 4000B system (Leica Microsystems, Wetzlar, Germany) under a 10× objective.

### 2.11 MTT assays

The toxicity of PLL/DNA/peptide polyplexes was evaluated by MTT assays. Briefly, HeLa cells, HEK293 cells U251 cells and COS7 cells were seeded in 96‐well plates (Corning Inc.) and transfected as described previously. After 24 hours of transfection, 20 μl of MTT solution (5 mg/ml) was added, and cells were incubated for 4 hours to allow the formation of formazan crystals. The medium was removed, and 150 μl of DMSO was added to each well to dissolve the formazan crystals. The absorbance was measured at 490 nm on a microplate reader (SpectraMax M5; Molecular Devices). Cell viabilities were calculated by:

$$\text{Viability}\;\left(\%\right)=\frac{A_{\text{treated}}-A_{\text{blank}}}{A_{\text{untreated}}-A_{\text{blank}}}\times 100\%$$

The mean ± SD of the absorbance was calculated for each group. Lipofectamine 2000 were used as a control group.

### 2.12 Statistical analysis

Statistical significance of the differences among groups was determined using unpaired Student's *t* tests.

## 3 RESULTS

### 3.1 Design of AMP‐derived endosomolytic peptides

Replacement of the positively‐charged residues of the naturally occurring membrane‐permeabilizing antimicrobial peptide with Glu yielded the pH‐sensitive endosomolytic peptides, which could enhance the PEI‐mediated transfection efficiency.^11^ To investigate the effect of Glu substituted for positively‐charged residues on the endosomal escape activity of AR‐23, the first positively‐charged residue of AR‐23(K13) was replaced by Glu(E) to generated aAR1 and aAR2 was designed by replacing K13 and K21 of AR‐23 with Glu. Finally, aAR3 was produced by substituting of K13, K21 and R23 in AR‐23 with Glu. The close agreement between the measured and theoretical molecular weights of the peptides suggesting that the peptides had been successfully synthesized (Table 1).

### 3.2 CD spectroscopic analysis of the peptides

**FIGURE 1:**
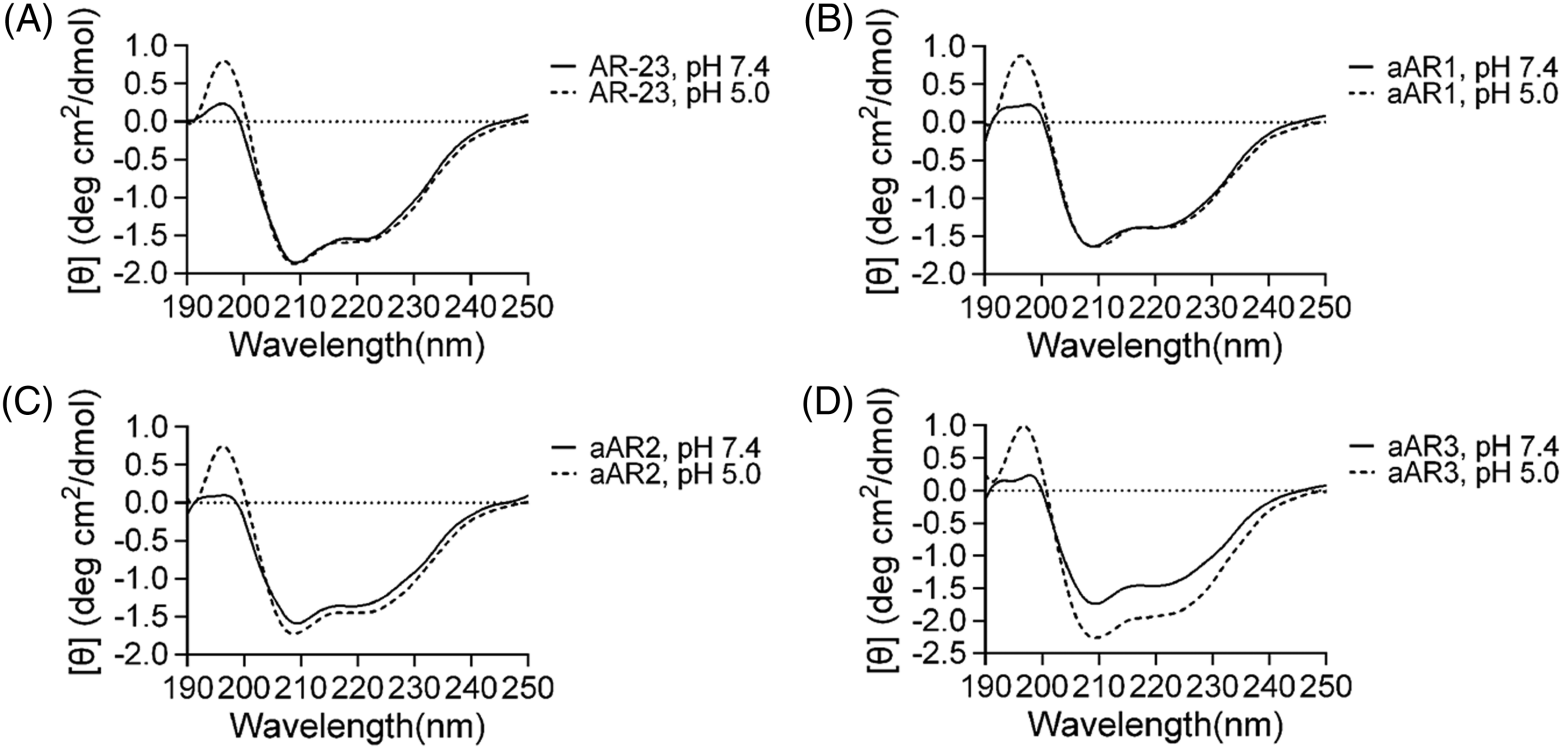
CD spectra of peptides acquired in 50% TFE at pH 5.0 and pH 7.4 at 25°C. All of the peptides formed a well‐defined α‐helical structure in the presence of 50% TFE. A, CD spectra of AR‐23; B, CD spectra of aAR1; C, CD spectra of aAR2; D, CD spectra of aAR3

The secondary structure of peptides in different pH environments (in 50% TFE pH 5.0 and pH 7.4) were determined by the CD spectra. As shown in Figure 1, all of the peptides exhibited a typical α‐helix spectrum with double minima at 208 nm and 222 nm in the two pH solutions with 50% TFE. Substitution of positively‐charged residues with Glu decreased the α‐helical content, and aAR2 exhibited the lowest α‐helical content of the four peptides at pH 7.4 (Table 2). Similar α‐helical content of AR‐23 at different pH was observed and so was aAR1, whereas the α‐helical content of aAR2 was slightly higher at pH 5.0 than that of at pH 7.4 and aAR3 possessed the highest α‐helical content of the four peptides at pH 5.0, which was 54.3%. The Gibbs free energy of the peptide partition from water to a membrane interface (Δ*G*_if_) was calculated by MPEx according to the helicities of the peptides at different pH levels^28^ (Table 2). Peptides at pH 5.0 exhibited lower Δ*G*_if_ compared to peptides at neutral conditions, whereas aAR3 possessed the highest Δ*G*_if_ at pH 7.4 and the lowest Δ*G*_if_ at pH 5.0.

**TABLE 2:** α‐Helical content and ΔG_if_ of the peptides

Peptide	% Helix in 50% TFE at pH 7.4 ^a^	% Helix in 50% TFE at pH 5.0 ^a^	Δ*G*_if_ pH 7.4 (kcal mol^–1^) ^b^	Δ*G*_if_ pH 7.4 (kcal mol^–1^) ^b^
AR‐23	44.0	44.6	−7.63	−7.72
aAR1	39.1	39.4	−6.14	−8.17
aAR2	37.9	41.1	−5.02	−9.35
aAR3	41.2	54.3	−4.08	−10.17

^a^ α‐helical content measured in 50% TFE at different pH levels.

^b^ Calculations of Δ*G*_if_ according to the helicities of the peptides at different pH levels were performed with MPEx.

### 3.3 pH‐dependent hemolytic activity

**FIGURE 2:**
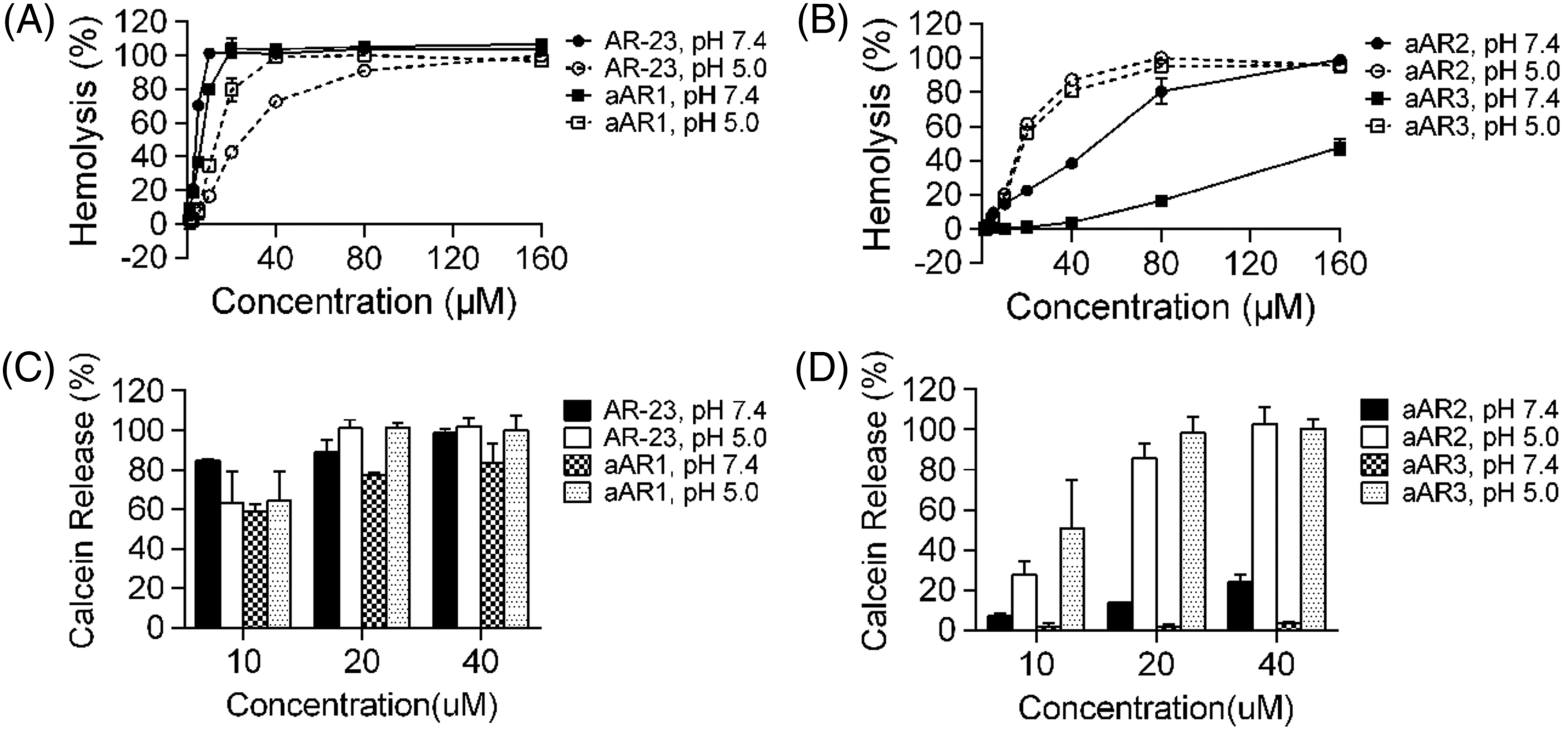
Hemolytic activity of peptides against human erythrocytes and comparison of calcein release by the peptides at pH 5.0 and pH 7.4. (A) Hemolytic activity of AR‐23 and aAR1. (B) Hemolytic activity of aAR2 and aAR3. (C) Calcein release induced by AR‐23 and aAR1. (D) Calcein release induced by aAR2 and aAR3. Each point represents the mean of three independent experiments with error bars indicating the SD

Glu replacement was sufficient to induce the pH sensitivity of AMPs. The impact of the number of Glu substituted for positively‐charged residues on the membrane lytic activity of AR‐23 was investigated by hemolytic assays at pH 5.0 and 7.4 using hRBCs. The hemolytic activity of AR‐23 and aAR1 at pH 7.4 was higher than that of them at pH 5.0, whereas aAR2 and aAR3 had an inverse tendency (Figure 2A and 2B). With the increasing number of glutamate residues, the hemolytic activities of the peptides were reduced at pH 7.4; for example, the hemolytic activity of AR‐23, aAR1, aAR2 and aAR3 at concentrations of 10 μM was 101.3%, 79.9%, 14.6 and 0.1%, respectively. The hemolysis rates of AR‐23 analogs at pH 5.0 were higher than that of AR‐23; for example, the hemolysis rates of AR‐23, aAR1, aAR2, and aAR3 were 42.7%, 79.7%, 61.4% and 56.0% at concentrations of 20 μm, respectively. It should be noted that the hemolysis rate of AR‐23 and aAR1 at pH 7.4 was higher than that of them at pH 5.0, whereas the hemolysis rates of aAR2 and aAR3 were lower than those under pH 5.0. A concentration of 40 μm aAR3 induced 80.3% cell lysis at pH 5.0, whereas only 3.8% cell lysis occurred at pH 7.4.

### 3.4 hRBC membrane integrity

Membrane permeabilization induced by the four peptides was measured by releasing entrapped calcein from hRBCs at pH 7.4 and pH 5.0. Similar to the results of the hemolytic assays, calcein release induced by peptides was reduced with an increasing number of the Glu at pH 7.4. aAR2 and aAR3 induced more calcein release at pH 5.0 (Figure 2C and 2D). For example, 20 μm aAR3 induced 98.4% and 1.9% calcein release at pH 5.0 and pH 7.4, respectively. On the other hand, AR‐23 and aAR1 induced higher calcein release at pH 7.4 than at pH 5.0.

### 3.5 Intracellular trafficking of the polyplexes

**FIGURE 3:**
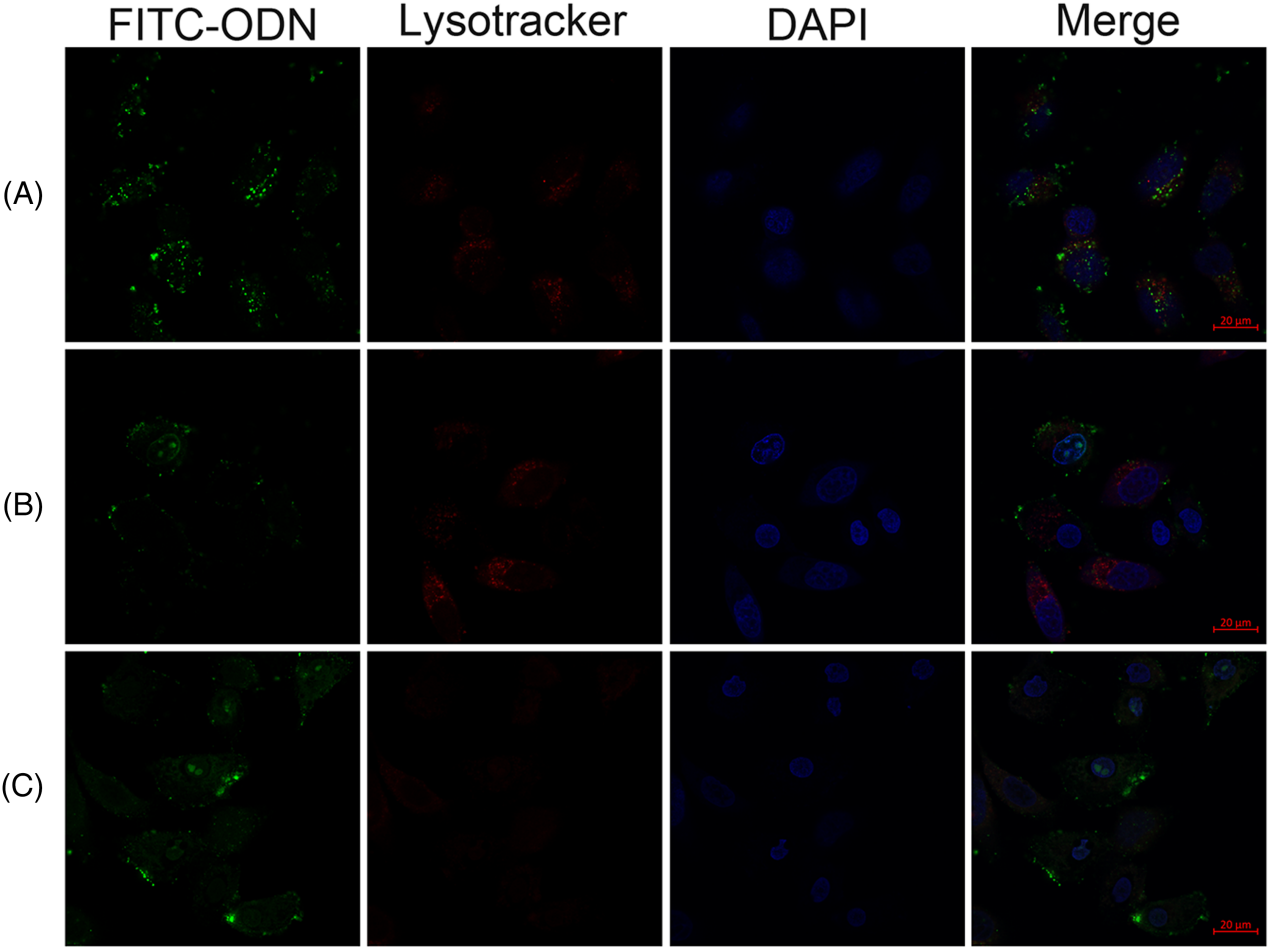
Confocal microscopy images of HeLa cells incubated with (A) PLL/DNA, (B) PLL/DNA with 2.5 μm AR‐23 and (C) PLL/DNA with 40 μm aAR3. Images were acquired with a confocal laser scanning microscope (scale bar = 20 μm) at an additional 1.5 hours after uptake and show the subcellular distribution of calcein fluorescence. The late endosomes and lysosomes are stained with Lyso‐tracker red, and cell nuclei are stained with DAPI

In the present study, FITC‐ODN was used to evaluate the endosomal lytic activity of the peptides. As shown in Figure 3, FITC‐ODN was restricted within intracellular vesicles and showed a punctate distribution of the fluorescence signal in the cytoplasm in the PLL/DNA‐only group. However, some PLL/DNA/aAR3‐treated and PLL/DNA/AR‐23‐treated cells showed dispersive green fluorescence signals in the cytoplasm, indicating late degradation of some endosomes/lysosomes. Moreover, some green fluorescence signal was also observed in the nuclei of PLL/DNA/AR‐23‐treated cells and PLL/DNA/aAR3‐treated cells. Furthermore, the PLL/DNA/aAR3‐treated cells showed stronger green fluorescence signals in the cytoplasm and nuclei than that of LL/DNA/AR‐23‐treated cells. The results indicated that aAR3 had a higher ability to promote the release of FITC‐ODN complexes from endosomes and subsequent transfer into the cytoplasm and nuclei, suggesting that the acidic peptides exhibited stronger endosomal lytic activity than AR‐23.

### 3.6 Physicochemical characteristics of the polyplexes

#### 3.6.1 DNA gel retardation assays

**FIGURE 4:**
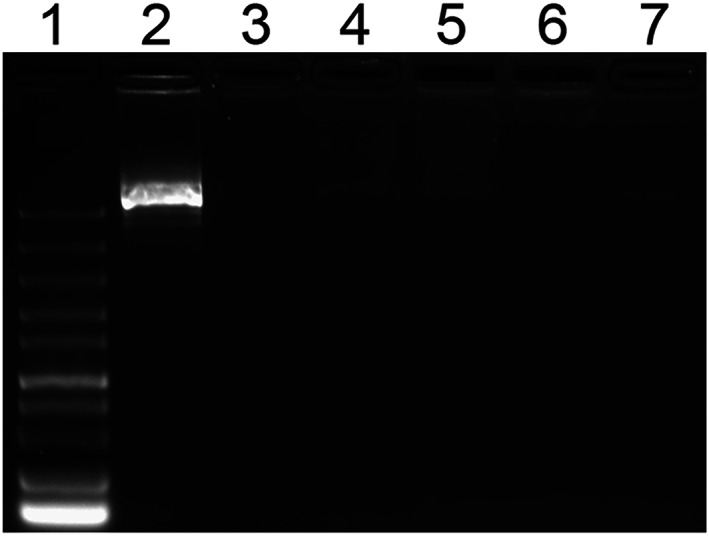
Agarose gel electrophoresis of PLL/DNA and PLL/DNA/peptide, the peptide was added at different concentrations as indicated in bracket. 1, Marker; 2, DNA (plasmid); 3, PLL/DNA; 4, PLL/DNA + AR‐23 (2.5 μm); 5, PLL/DNA + aAR1 (10 μM); 6, PLL/DNA + aAR2 (20 μm); 7, PLL/DNA + aAR3 (40 μm)

To investigate the effect of AR‐23 and its analogs on the capability of PLL to condense plasmid DNA, a DNA gel retardation experiment was performed for different concentrations of peptides mixed with PLL/DNA polyplexes. Free plasmid DNA was included as a control. Compared to control plasmid DNA, almost no migrated band was observed for PLL/DNA polyplexes or PLL/DNA polyplexes mixed with peptides (Figure 4). The results suggested that incorporation Glu‐replaced AR‐23 does not affect the binding ability of PLL to plasmid DNA.

#### 3.6.2 Size and zeta potential of the polyplexes

**FIGURE 5:**
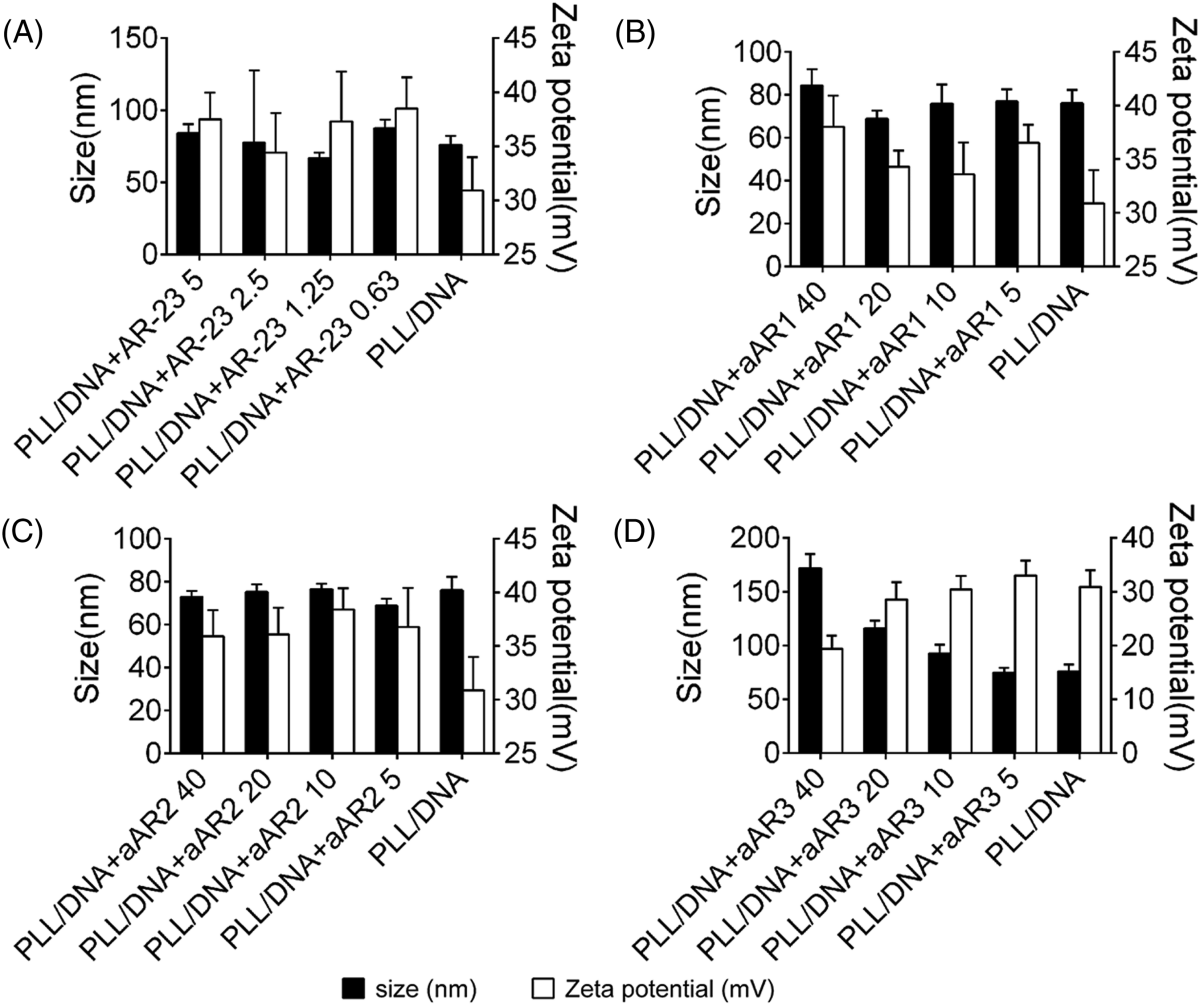
Particle sizes and zeta potentials of PLL/DNA and PLL/DNA/peptide. The N/P ratio of PLL/DNA was 5, and the peptide was added at different concentrations as indicated behind the peptide. A, particle sizes and zeta potentials of PLL/DNA and PLL/DNA/AR‐23; B, particle sizes and zeta potentials of PLL/DNA and PLL/DNA/aAR1; C, particle sizes and zeta potentials of PLL/DNA and PLL/DNA/aAR2; D, particle sizes and zeta potentials of PLL/DNA and PLL/DNA/aAR3

To investigate the effects of peptides on the physicochemical characteristics of PLL/DNA polyplexes, the size and zeta potential of the polyplexes were measured by dynamic light scattering. The size of the PLL/DNA polyplexes (N/P = 5) was 76 nm (Figure 5). Generally, minor changes in the size of the PLL/DNA polyplexes were observed when AR‐23, aAR1 or aAR2 were incorporated (Figure 5). For example, the average size of PLL/DNA/AR‐23, PLL/DNA/aAR1 and PLL/DNA/aAR2 polyplexes was mainly distributed between 65–85 nm. However, the average size of PLL/DNA/aA23 polyplexes increased with increasing peptide concentrations. For example, the average sizes of the PLL/DNA polyplexes were 171.7 nm and 116.1 nm when 40 μm and 20 μm aA23 were incorporated (Figure 5D).

The zeta potential of the PLL/DNA polyplexes also slightly changed as a result of the incorporation of AR‐23, aAR1or aAR2 at different peptide concentrations. However, significant changes were observed when aAR3 were incorporated into the PLL/DNA polyplexes at different peptide concentrations (Figure 5). For example, the zeta potential of the PLL/DNA/AR‐23, PLL/DNA/aAR1 and PLL/DNA/aAR2 polyplexes primarily ranged from 30 to 40 mV. The zeta potential of the polyplexes decreased with increasing amounts of aAR3.

### 3.7 Transfection efficiency assay

**FIGURE 6:**
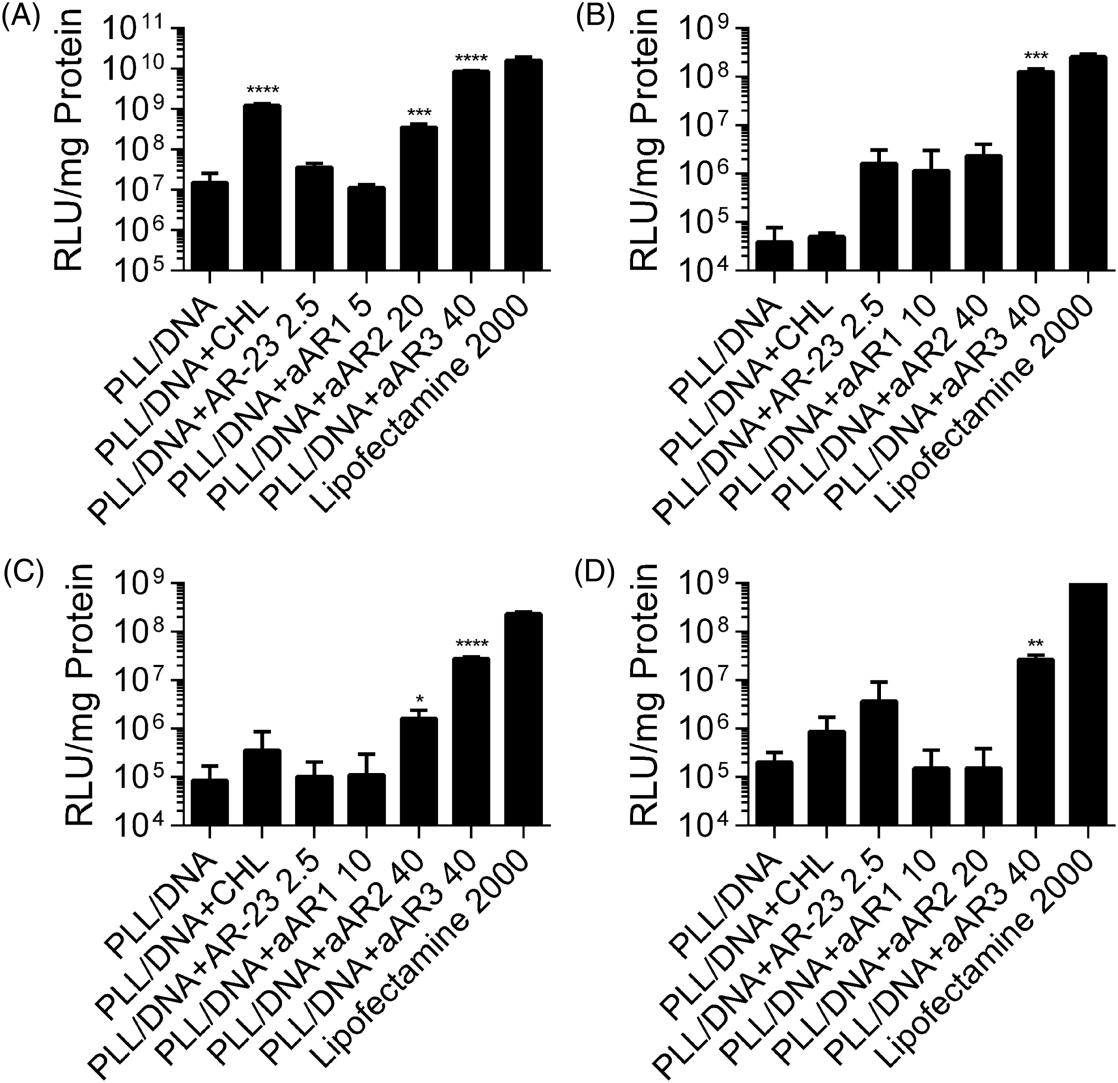
Comparison of the transfection efficiency of PLL/DNA and PLL/DNA/peptides in (A) HEK‐293 cells, (B) Cos7 cells, (C) U251cells and (D) HeLa cells. Chloroquine (CHL) and Lipofectamine 2000 were used as positive control. Data are the mean ± SD (*n* = 3). *p < 0.05, **p < 0.01, ***p < 0.001 and ****p < 0.0001 as determined by an unpaired Student's *t* test

**FIGURE 7:**
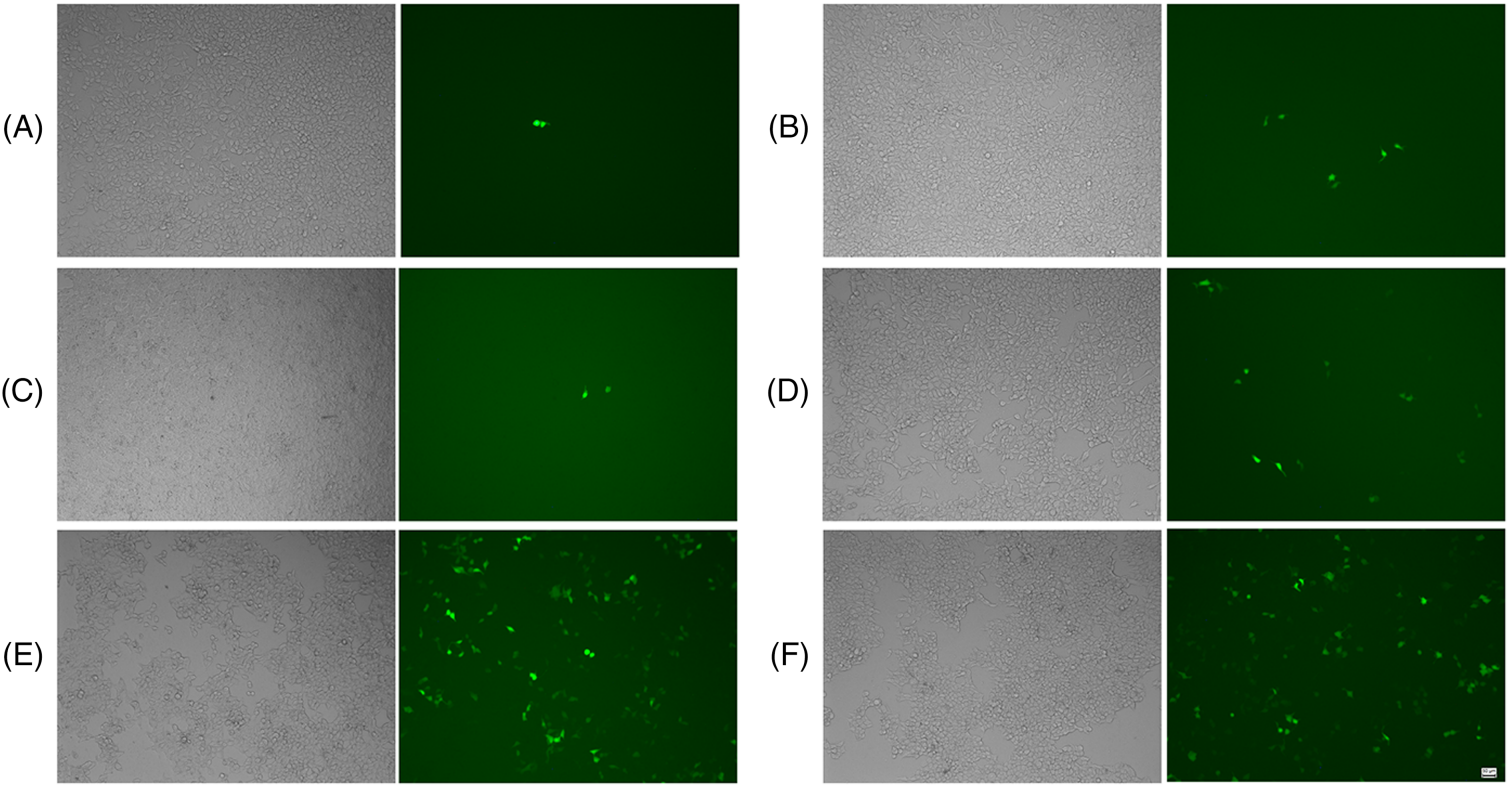
Transfection efficiency of PLL/DNA and PLL/DNA/peptides in HEK‐293 cells, images were acquired with an inverted fluorescence microscope (scale bar = 50 μm). (A) PLL/DNA; (B) PLL/DNA + AR‐23 (2.5 μm); (C) PLL/DNA + aAR3 (5 μm); (D) PLL/DNA + aAR2 (20 μm); (E) PLL/DNA + aAR3 (40 μm); and (F) Lipofectamine 2000

Luciferase plasmid was used as a reporter gene to investigate the effect of peptides on PLL‐mediated gene transfection in four different cell lines (HEK‐293, U251, Cos7 and HeLa). AR‐23 and aAR1 slightly increased the transfection efficiency of the PLL/DNA polyplexes in the four cell lines (Figure 6). The optimal concentrations of aAR2 and aAR3 for achieving the highest enhancement of PLL‐mediated transfection efficiency were varied in different cell lines. A dramatic enhancement of luciferase gene expression was observed upon incorporating the aAR2 and aAR3 for all the tested cell lines, and PLL/DNA/aAR3 polyplexes generated the highest transfection efficiency. The highest transfection efficiencies of the PLL/DNA/aAR3 polyplexes were 565.2‐, 719.5‐, 3,268‐ and 129.2‐fold of the PLL/DNA polyplexes in HEK‐293 cells, U251 cells, Cos7 cells and HeLa cells, respectively. Chloroquine, a reagent commonly used to promote the release of polymers from endosomes, could also increase the transfection efficiency of the PLL/DNA polyplexes in the four cells. In the presence of chloroquine, PLL‐mediated gene transfection efficiency was increased by 81.9‐, 9.2‐, 1.3‐ and 4.2‐fold in HEK‐293 cells, U251 cells, Cos7 cells and HeLa cells, respectively. The results indicated that Glu‐substituted peptides could enhance PLL‐mediated transfection. Furthermore, PLL/DNA/aAR3 polyplexes induced comparative EGFP expression to the Lipofectamine 2000 group (Figure 7).

### 3.8 Toxicity of PLL/DNA/peptide polyplexes

**FIGURE 8:**
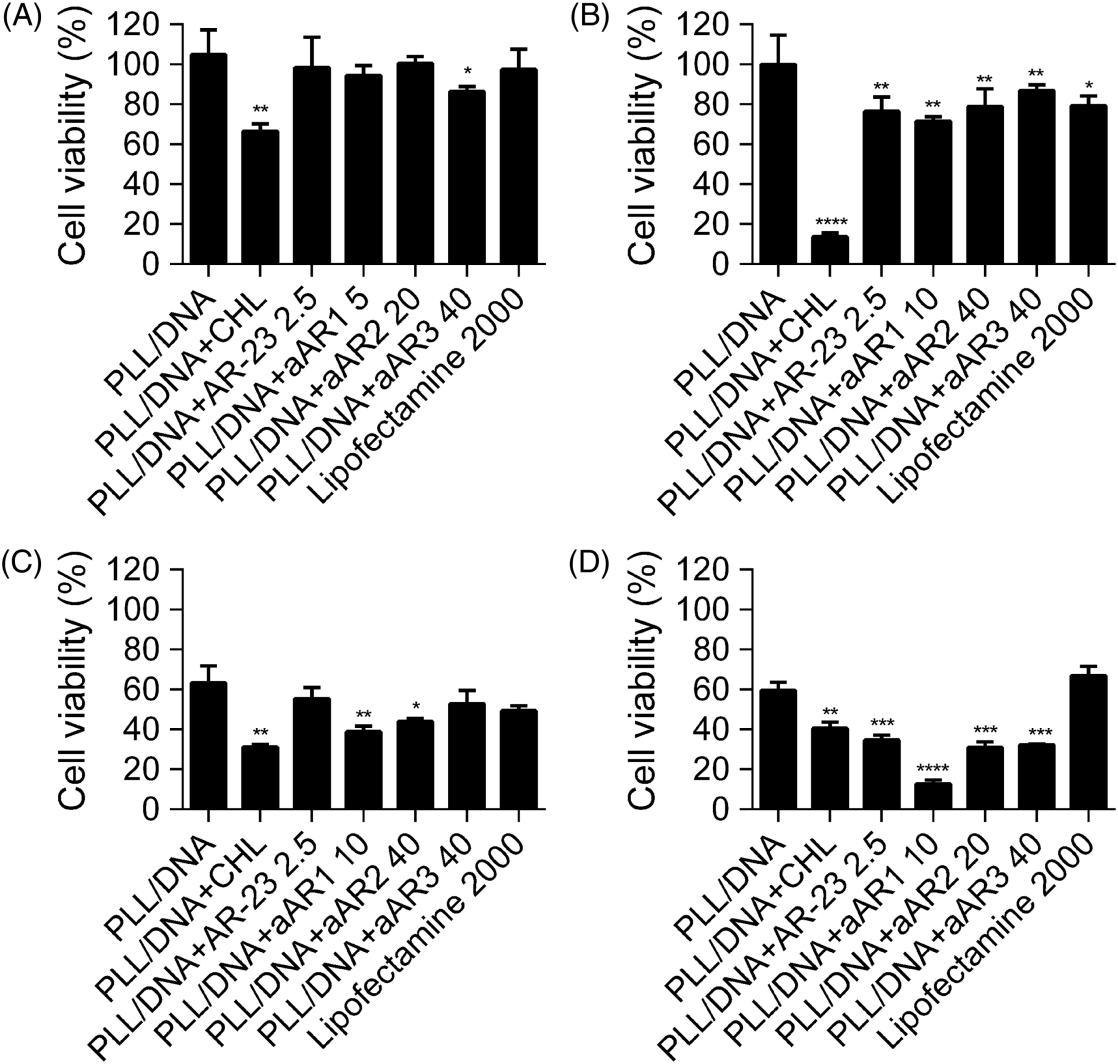
Cytotoxicity of PLL/DNA and PLL/DNA/peptide in (A) HEK‐293 cells, (B) U251 cells, (C) Cos7 cells and (D) HeLa cells. Data are the mean ± SD (*n* = 3). *p < 0.05, **p < 0.01, ***p < 0.001 and ****p < 0.0001 as determined by an unpaired Student's *t* tests

The inherent membrane lytic activity of AMPs prompted us to further evaluate the cellular toxicity of the PLL/DNA/peptides polyplexes. The cytotoxicity of the polyplexes was determined by MTT assays in HEK‐293 cells, U251 cells, Cos7 cells and HeLa cells. The results showed that the incorporation of Glu‐substituted peptides into the PLL/DNA polyplexes increased cytotoxicity in the four cell lines (Figure 8). For example, the cell viability of U251 cells upon treatment with the PLL/DNA polyplexes was 63.3%, and incorporation of 40 μm aAR3 into the PLL/DNA polyplexes decreased the cell viability to 52.8%. However, the cell viability of U251 treated with PLL/DNA polyplexes incorporated with 40 μm aAR3 was still higher than that of U251 treated with Lipofectamine 2000.

## 4 DISCUSSION

Viruses invade live cells via receptor‐mediated endocytosis, and the internalized virus is trafficked to late endosomes.^29^ The acidic endosomal environment induces membrane fusion between the virus and endosomes via a conformational change of surface proteins, and the viral genome is released into the cytoplasm of the target cells, thus enabling escape from the endosomes.^30^ The major envelope protein of the West Nile virus is another example of a fusogenic agent that exerts its endosomal disruptive activities at an upper threshold of pH 7.0 and has a maximum activity at pH 6.4 and below, which leads to maximum conformational change in the protein within seconds.^31^ Several peptides derived from viral proteins also have been shown to exert endosomolytic activity. These peptides are enriched in glutamic acids, which may be related to their lytic activity at acidic pH. GALA and INF7 are glutamic acid‐rich peptides that show significant toxicity at pH 5.0 but negligible toxicity at similar concentrations at pH 7.0.^32^, ^33^ Glu is partially protonated at pH 5.0, which appears to be sufficient for inducing the pH responsiveness of these peptides.^17^, ^18^, ^34^ Replacement of the positively‐charged residues of the naturally occurring membrane‐permeabilizing antimicrobial peptide with Glu yielded the pH‐sensitive endosomolytic peptides, which could enhance the PEI‐mediated transfection efficiency.^11^

The present study aimed to investigate the effect of the number of glutamic acids substituted for positively‐charged residues on the endosomal escape activity of AR‐23 and the ability of modified AR‐23 for enhancing cationic polymer mediated transfection. Glu replacement decreased the α‐helical content of AR‐23 in 50% TFE at pH 7.4 (Table 2); however, aAR3 showed higher α‐helical content in 50% TFE at pH 5.0 than that at pH 7.4. The low Gibbs free energy of the peptide partition from water to a membrane interface (Δ*G*_if_) indicates a strong membrane partition.^28^ An increased in the Δ*G*_if_ of peptides was observed with the an increased number of Glu replacements in the AR‐23 sequence at pH 7.4, indicating that Glu replacement decreased the binding ability of the peptides with eukaryotic membranes at neutral pH (Table 2). Quite an opposite tendency was observed at acidic pH. Indeed, AR‐23 is an AMP with high lytic activity at neutral pH. A concentration of 5 μm AR‐23 induced 70% and 6.5% hRBC lysis at pH 7.4 and pH 5.0, respectively, which means that AR‐23 was a toxic peptide with low endosomal disrupting activity. However, after Glu replacement, the hemolysis rate of various analogs at pH 7.4 was greatly reduced, especially aAR3. Membrane permeabilization induced by the four peptides were similar to the results of the hemolytic assays at pH 7.4, and calcein release and hRBC lysis induced by peptides were both reduced with an increasing number of the glutamate residues. However, calcein release and hRBC lysis induced by peptides were comparable at pH 5.0. The above data suggested that aAR3 induced significantly more hRBC membrane disruption at pH 5.0 than at pH 7.4, which allows pH‐sensitive lysis of cell membranes and exertion of endosomolytic activity at acidic pH.

PLL/DNA polyplexes are primarily taken up through endocytosis. Eventually, the polyplexes are enclosed in the low‐pH environment of endosomes for degradation by hydrolytic enzymes. FITC‐ODN was used to evaluate the intracellular trafficking of the polyplexes and the endosomal lytic activity of acidic peptides. As shown in Figure 3, most of the FITC‐ODN was restricted within intracellular vesicles and showed a punctate distribution in the PLL/DNA‐only group. However, dispersive green fluorescence signals were observed in the PLL/DNA/aAR3‐treated cells, which indicates the degradation of some late endosomes/lysosomes (Figure 3C and 3D). Moreover, green fluorescence signals were observed in the nuclei of some PLL/DNA/aAR3‐treated cells. The results indicated that aAR3 had strong endosomal lytic activity, which could promote FITC‐ODN release into the cytoplasm and subsequent transfer to nuclei.

The physicochemical characteristics of the polyplexes, such as the size and the zeta potential of the polyplexes, play pivotal roles in determining the gene delivery efficiency both *in vitro* and *in vivo.* ^35^ In the present study, DNA gel retardation assays indicated that the glutamate residues number of AR‐23 would not affect the binding ability of PLL to plasmid DNA (Figure 4) and the particle size of the polyplexes only increased with increasing of aAR3 incorporation (Figure 5). Because larger‐sized PLL/DNA polyplexes showed higher transfection efficiency than smaller‐sized polyplexes,^36^, ^37^ the appropriate amount of peptides would positively affect the particle size for gene transfection. On the other hand, the zeta potential of the polyplexes was affected slightly by the amount of acidic peptides. The zeta potential of the PLL/DNA/AR‐23, PLL/DNA/aAR1 and PLL/DNA/aAR2 polyplexes was increased slightly with differing amounts of peptides incorporation, and 40 μm aAR3 incorporation to the PLL/DNA/polyplexes decreased its zeta potential (Figure 5). Because triple‐Glu substituted peptide, aAR3, is negatively charged in water, it would electrostatically interact with the positively‐charged PLL/DNA polyplexes, thus decreasing its zeta potential.

The ability of the peptides to enhance gene transfer of PLL/DNA polyplexes was tested in four cell lines (HEK‐293, U251, Cos7 and HeLa). No significant enhanced transfection efficiency was observed with the incorporation of various concentrations of AR‐23 and aAR1 into PLL/DNA polyplexes in the four cell lines; furthermore, high concentrations of AR‐23 and aAR1 incorporation induced unacceptable cytotoxicity. As noted above, aAR2 and aAR3 were more lytic at pH 5.0, and the incorporation of aAR2 and aAR3 into PLL/DNA polyplexes enhanced the transfection efficiency in the four cell lines, especially aAR3. (Figure 6). In all of the tested cell lines, 40 μm aAR3 incorporation to the PLL/DNA/polyplexes generated the highest transfection efficiency without causing remarkable cytotoxicity. The highest transfection efficiencies of the PLL/DNA/aAR3 polyplexes were 565.2‐, 719.5‐, 3,268‐ and 129.2‐fold of the PLL/DNA polyplexes in HEK‐293 cells, U251 cells, Cos7 cells and HeLa cells, respectively. The results indicated that the number of glutamic acids substituted for positively‐charged residues of AR‐23 dramatically affects its ability for enhancing PLL‐mediated transfection. The impact of endo/lysosomal escape on transgene expression was investigated by transfecting cells in the presence of chloroquine, a reagent commonly used to promote the release of polymers from endosomes.^38^, ^39^ In the presence of chloroquine, the highest transfection efficiency of PLL/DNA polyplexes was achieved in HEK‐293 cells. However, aAR3 enhances PLL‐mediated gene transfection to a greater extent than chloroquine. Furthermore, PLL/DNA/aAR3 polyplexes induced comparative luciferase activity and cytotoxicity to the Lipofectamine 2000 group. The above results indicated that the incorporation of the triple‐Glu substituted peptide could promote appreciable disruption of the endosomal membrane and promote the entry of polyplexes into the cytoplasm, thus increasing transfection efficiency.

## 5 CONCLUSIONS

In summary, the increased lytic activity of peptides was observed with an increased number of Glu replacements in the AR‐23 sequence at acidic pH. The number of glutamic acids substituted for positively‐charged residues of AR‐23 dramatically affects the ability to enhance PLL‐mediated transfection. Triple‐Glu substitution in the AR‐23 sequence improved lytic ability at acidic pH and decreased cytotoxicity at neutral conditions. The designed aAR3 interacted with PLL/DNA polyplexes and increased the particle size of the polyplexes. aAR3 showed higher endosomolytic activity and greatly improved PLL‐mediated gene transfection efficiency at the same time as maintaining low cytotoxicity. We suggest that sufficient glutamic acid residue replacement may be considered as a method for designing pH‐sensitive peptides, which could be applied as potential enhancers for improving the transfection efficiency of cationic polymers. However, more work is required to achieve synthetic virus‐like particles that can transfect genes into specific cells in an efficient and safe manner.

## AUTHOR CONTRIBUTIONS

ZSK, GL, YZM and ZX performed the research. ZSK, GL and JSP designed the research study. GHW, LSB, DCJ and YJJ collected and analyzed the data. ZSK, CJM and TYX wrote the paper. GF and JSP supervised the project and revised the manuscript. All authors read and approved the final manuscript submitted for publication.

## CONFLICT OF INTEREST STATEMENT

The authors declare that they have no conflicts of interest.

## Data Availability

All data and materials regarding the study are available from the corresponding author upon reasonable request.
